# Sticks and Stones: Hand Preference and Grip in Wild Chimpanzee Tool‐Use

**DOI:** 10.1002/ajpa.70364

**Published:** 2026-10-10

**Authors:** Ammie K. Kalan, Mimi Arandjelovic, Hjalmar Kuehl, Skye Bartel‐Ens, Amy Christensen, Julia Whelan, Anthony Agbor, Paula Dieguez, Juan Lapuente, Amelia C. Meier, Fotios Alexandros Karakostis

**Affiliations:** ^1^ GAB Lab, Department of Anthropology University of Victoria Victoria British Columbia Canada; ^2^ DFG Center ‘Words Bones Genes Tools’ University of Tübingen Tübingen Germany; ^3^ Department of Primate Behavior and Evolution Max Planck Institute for Evolutionary Anthropology Leipzig Germany; ^4^ German Centre for Integrative Biodiversity Research (iDiv) Halle‐Jena‐Leipzig Leipzig Germany; ^5^ Senckenberg Museum for Natural History Görlitz, Senckenberg – Member of the Leibniz Association Görlitz Germany; ^6^ International Institute Zittau Technische Universität Dresden Zittau Germany; ^7^ Jane Goodall Institute Washington DC USA; ^8^ Max Planck Institute for Evolutionary Anthropology Leipzig Germany; ^9^ Palaeoanthropology, Institute for Archaeological Sciences, Department of Geosciences University of Tübingen Tübingen Germany; ^10^ Senckenberg Centre for Human Evolution and Palaeoenvironment Senckenberg Research Institute Tübingen Germany; ^11^ Integrative Prehistory and Archaeological Science University of Basel Basel Switzerland

## Abstract

**Objectives:**

Humans exhibit a near‐universal right‐handed bias, linked to hemispheric brain lateralization, yet its evolutionary origins remain unclear. Wild chimpanzees, one of our closest living relatives, show diverse tool‐use behaviors and substantial variability, providing an appropriate context to investigate the emergence and functional significance of handedness in ecologically relevant settings. This study aimed to assess chimpanzee handedness and grip at both the individual and behavioral levels to determine whether specific tool‐use tasks elicit consistent preferences.

**Methods:**

We investigated hand preferences and grasping patterns in wild chimpanzees for three tool‐use behaviors observed in three different communities: accumulative stone throwing, algae fishing, and nut cracking. Our dataset consisted of camera‐trap videos of unhabituated wild chimpanzees with individuals identified across videos.

**Results:**

We found 20/21 individual chimpanzees were consistent in their hand preference during primary tool‐use actions: fishing, hammering, or throwing. Insufficient observations of a further 16 chimpanzees prevented assessment of their hand preference. We found no evidence for behavior‐ or population‐level biases in hand preference. Grips were only possible to code for algae fishing, where we found pronounced inter‐individual variability in grasping, with notable trends between left‐ and right‐handed chimpanzees. Also, right‐handed individuals appeared to fish for longer durations, although the implications for tool‐use performance remain unclear.

**Discussion:**

This study highlights challenges and complexity associated with plant‐based precision‐grasping tool‐use behaviors like algae fishing. During the early stages of hominin evolution, the efficient execution of such manual behaviors likely benefited from key evolutionary adaptations favoring improved dexterity, motor skills, and enhanced brain‐hand coordination.

## 1 Introduction

The near universal right‐hand dominance observed in human populations has puzzled scholars for centuries (McManus 2019; Prieur et al. 2018). Moreover, this asymmetry in hand use is further reflected in the lateralization of our brain (Fitch and Braccini 2013; McManus 2019; Stout and Chaminade 2012). Countless studies have shown that there are intricate neurobiological relationships between the hemispheric specializations of our brain with hand‐eye motor coordination and language development (Becker et al. 2022; Fagot and Vauclair 1991; Hopkins et al. 2019; McManus 2019; Prieur et al. 2019; Stout and Chaminade 2012). Given the significance of both tool technology and language in the course of human evolution, the lateralization of the human brain and when in our evolutionary history this emerged has been the focus of much research (Cashmore et al. 2008; Cochet and Byrne 2013; Kalan 2023). Do nonhuman primates exhibit hemispheric specialization and if so, at which point in the Primate Order does this appear? Or, is lateralization unique to humans, potentially emerging in hominins after the *Pan‐Homo* lineages split? Given the invasive nature of directly measuring cerebral asymmetry, handedness has been used for decades as a proxy for lateralization. The tendency to naturally use the left or right hand more in humans and other primates has been investigated both in the lab and in more natural settings, with much of the literature focused on experimental studies (Hopkins 1996; Hopkins et al. 2011; Kalan 2023; Meguerditchian et al. 2015; Prieur et al. 2018). However, despite a number of handedness studies on wild chimpanzees in tool use, foraging and communicative contexts, there is no clear consensus on the existence, or lack thereof, of a population‐level handedness bias (Boesch 1991; Bogart et al. 2012; Hobaiter and Byrne 2013; Humle and Matsuzawa 2009; Lonsdorf and Hopkins 2005; McGrew et al. 1999; McGrew and Marchant 1992, 1999; Nishida et al. 2012; Sanz et al. 2016; Sugiyama et al. 1993).

Comparative methodological approaches provide a robust foundation for reconstructing the manual behaviors of extinct fossil hominin species (Cashmore et al. 2008). They also shed light on the factors that may have driven the emergence of the uniquely high thumb‐based precision grasping efficiency observed in modern humans, an ability that enables skillful manipulation of small objects within the palm and fingers (Karakostis, Haeufle, et al. 2021; Kivell et al. 2023; Kunze et al. 2024; Marzke et al. 2015; Marzke and Wullstein 1996; McGrew and Foley 2009; Tennie et al. 2017). This evolutionary framework is particularly relevant for the great apes and their tool‐use behaviors, given the fundamental importance that tool manufacture and use has had throughout hominin evolution (Cashmore et al. 2008; Stout et al. 2008). In this regard, the manual behavior and hand preference patterns observed in chimpanzees, are of key significance to constructing evolutionary models linking relationships between handedness and tool use throughout human evolution (Cochet and Byrne 2013; Marzke and Wullstein 1996; Uomini 2009). In the wild, chimpanzees naturally show the most diverse tool use behaviors and employ the most diverse tool use materials of any animal, surpassed only by humans (Kalan et al. 2020).

In this study, we provide new insights into the manual behaviors of wild chimpanzees when handling and using sticks and stones as tools. Specifically, we focus on three tool‐using behaviors that have recently been described for wild chimpanzees but have not yet been the subject of detailed studies on hand use, including the precise manipulation involved in algae fishing (Boesch et al. 2017) and forceful, accumulative stone throwing (AST; Kühl et al. 2016). Additionally, we had the opportunity to examine stone hammer nut cracking data from populations that have been underrepresented in the literature.

Our first objective was to assess hand preference at both the individual and behavior‐level, to determine if any bias to the left or right exists for certain tool‐use tasks. Evidence for sex differences in wild chimpanzee hand preference is limited, with only termite fishing demonstrating a sex bias in hand use whereby females are more likely to be left‐handed (Sanz et al. 2016). Therefore, at the individual‐level we expected to see a lot of variation with no clear expectation for the data other than potential sex differences, given that in humans left‐handedness has been shown to be more prevalent among males (Papadatou‐Pastou et al. 2008). At the behavior‐level, right and left‐handed bias have been documented for different chimpanzee tool‐use behaviors in the wild (Kalan 2023; Marchant 2015). Nut‐cracking has shown a right‐hand bias (Lonsdorf and Hopkins 2005) but see also (Boesch 1991; Sugiyama et al. 1993) and termite‐fishing a left‐hand bias (Bogart et al. 2012; McGrew and Marchant 1992) but see also (Sanz et al. 2016). A right‐hand bias has also been reported for ant dipping (Humle and Matsuzawa 2009). A previous study on handedness in directed throwing behavior showed no clear bias (Nishida et al. 2012). We therefore predicted that nut cracking may show a right‐hand bias and that algae fishing, being most similar in its actions to other stick tool‐use behaviors such as ant‐dipping and termite‐fishing, could show a bias in either direction. However, in a study on arguably the most closely related behavior to algae fishing, algae scooping, no handedness bias was found (Humle and Matsuzawa 2009). With respect to AST, we expected no clear bias as was found in previous work by Nishida et al. (2012).

Our second objective was to describe the grasping patterns involved in algae fishing and nut cracking. Grip was not assessed for AST as the throwing behavior occurs too quickly with low resolution. Unfortunately, our nut‐cracking videos were too dark to permit reliable grip analysis and lacked sufficient repeats per individual to allow for detailed assessment of interindividual variability. Therefore, grip data are reported for algae fishing only. Algae fishing requires a structured series of fine manipulatory steps that may share similarities with certain types of human‐like precision grasping, particularly when manipulating a small cylindrical handle using the palm and/or fingers. Similar to hand preference, we anticipated that the grasping patterns would largely resemble those seen in other wild chimpanzee behaviors involving sticks, such as termite fishing (Lesnik et al. 2015; Osuna‐Mascaró et al. 2021). Furthermore, given the various genetic and/or environmental factors proposed to influence grasping performance in chimpanzees (e.g., Hopkins et al. 2019), we anticipate notable inter‐individual variation in grasping patterns that is not necessarily associated with hand preference and/or sex, along with within‐individual consistency across different algae‐fishing events.

## 2 Methods

### 2.1 Data Collection

Video data of wild chimpanzee tool use behavior were leveraged from the Pan African Program: the Cultured Chimpanzee (PanAf) (Kühl et al. 2019) from three different field sites in West Africa where chimpanzee communities were not habituated for research at the time of data collection. The PanAf data include nut cracking from two rainforest sites (Sapo, Liberia in 2011–2012 and Djouroutou, Côte d'Ivoire in 2012–2013) and algae fishing from a savanna woodland site (Bakoun, Guinea in 2014–2015). At all three sites, Bushnell Trophy HD camera trap devices were installed for a period of 12–16 months, with a combination of systematic and targeted sampling focused on chimpanzee behavior. Camera‐traps were set to record 1‐min video clips whenever the infrared sensor detected movement in front of the lens. At the fourth site, Boe in Guinea‐Bissau, camera‐trap data were collected in 2017 by AK for a previous project investigating AST behavior (Kalan, Carmignani, et al. 2019). The same models of Bushnell Trophy HD camera traps were installed to monitor 12 AST sites for 5 months. Camera traps were set to record 1‐min video clips as with the PanAf. To the best of our knowledge, each of the chimpanzee communities studied engage in only one of the tool use behaviors examined in this study; therefore, it was not possible to examine more than one of these behaviors per site.

### 2.2 Video Data Processing

The full camera trap video data from each site was processed by the community science platform Chimp&See for the three PanAf sites (Arandjelovic et al. 2024). All videos that were classified and/or tagged by community scientists as (1) having at least one chimpanzee visible and (2) relevant for tool use were included in this study. Given that only one tool use behavior dominated observations for each of the three sites, this permitted us to use this sample for more detailed coding. For Boe, all videos with a chimpanzee were identified in the field. This was facilitated by the small number of observations and targeted recordings at AST sites only.

Only videos where individual identification of the tool user was possible were used for this study. Chimpanzees were assigned sex and age classes (adult, adolescent, juvenile or infant) based on their size and genitalia (Goodall 1986). Individual identification was facilitated by facial features, injuries, and other unique bodily characteristics or patterns. For the three PanAf sites, individual identification was done by the community scientists on Chimp&See following standard methods (Debetencourt et al. 2023; McCarthy et al. 2021). For the Boe videos, individual identification was first done by JW, who had no experience with the dataset. All individual assignments were assessed by AK, an expert on chimpanzee behavior, in consultation with JW. Only individuals where JW and AK agreed upon their identification were included in this study.

To account for the lack of independence of all observations, we assigned event IDs to all videos. This was important given that chimpanzees travel in small subgroups or parties and the camera can be repeatedly triggered to record when parties spend considerable time at the same place, such as during tool use activities. Consecutive camera trap videos from the same place and day where the time difference was less than 15 min were considered the same behavioral event and given the same event ID. The use of event ID to control for repeated video observations from the same chimpanzee party has been effectively applied in previous camera trap video analyses (Kalan, Hohmann, et al. 2019; McCarthy et al. 2021).

### 2.3 Behavior Coding and Laterality

Laterality and grip patterns take time to develop but are expected to be established in individuals by adolescence (Boesch 1991; Humle and Matsuzawa 2009; Malherbe et al. 2024) therefore analyses were restricted to adolescents and adults (minimum estimated age of approximately 13+ years). These individuals were also possible to sex from video footage therefore permitting the analysis of potential sex differences. To code tool use behavior and handedness, SB‐E, AC, and JW coded the selected camera trap videos from each site using the program BORIS (Friard and Gamba 2016). The software permits precise annotations of a customized ethogram whereby data are exported into csv format for analysis. Our ethogram included coding which hand(s) used for primary tool use action (i.e., fishing, hammering, or throwing). We also noted which hand(s) were used to pick up tools whenever this was observed but did not examine this further due to the low number of observations (this aspect often happened off screen or out of view). If the primary tool use action was not clearly observable on screen the video was not coded (i.e., video cut off before chimp actually started fishing or chimpanzee only inspecting or carrying tools but not using them on camera).

The start and stop of each tool use episode (algae fishing or nut cracking) were also coded. For algae fishing, the start of an episode was anytime the end of the stick entered the water, and stop was once the end of the stick exited the water. For nut cracking, the start of an episode was when the hammer was at maximum point in the air before being brought down in the first strike, and stop was when the nut had been cracked open, that is, the chimp started to eat the pounded remains, or when the pounding stopped and the chimpanzee walked away. Note, there could be multiple episodes in a single camera‐trap video. Episode duration was automatically calculated by BORIS using the start and stop timestamps of each episode. Tool use episodes were always associated with a start time but would be coded as incomplete if the stop behaviors were not observed in the same video. Start and stop were irrelevant for AST because each throw is rather instantaneous. Each behavioral event or event ID could therefore be comprised of multiple episodes from the same chimpanzee as well as other chimpanzees present in the party (see Data S1).

To test the reliability of our coding, an independent research assistant, naïve to the study aims and hypotheses, re‐coded a random selection of nut cracking and algae fishing videos using the same BORIS program and ethogram. Videos were randomly selected, balanced across individual chimpanzees and events to ensure broad representation of the data. Due to the small AST dataset, JW first coded the videos for hand used and AK independently coded all the data for hand used during throws to examine agreement. The type of tool use behavior (picking up tool, fishing, hammering) and the hand used during fishing or hammering episodes was compared using the Cohen's Kappa test for measuring inter‐rater agreement from the function kappa2 from the R package “irr”. The timing for the start and the stop of nut cracking or algae fishing episodes was compared by calculating whether the difference between the timestamps of both coders for the same episode was significantly different than expected by chance. This was calculated using a permutation test across randomized samples of time differences with 1000 permutations for start and stop times separately.

### 2.4 Grip Coding During Algae Fishing

The descriptions of grasping patterns during algae fishing events were carried out by one of the authors (FAK), who has expertise in the characterization of primate manual behavior (e.g., Eteson et al. 2024, 2025; Karakostis et al. 2018; Karakostis, Haeufle, et al. 2021; Karakostis, Reyes‐Centeno, et al. 2021; Kunze et al. 2024). We relied on terminology and descriptions established in previous literature (Lesnik et al. 2015; Marzke et al. 2015; Marzke and Wullstein 1996; Osuna‐Mascaró et al. 2021), which were enriched with additional observations and insights for this study's specific context. A total of 41 videos capturing discernible fishing events from 10 individuals were analyzed, selected based on offering a clear and unobstructed view of the hands during fishing, while multiple other videos/events had to be excluded due to insufficient resolution and/or inappropriate viewing angles. Unfortunately, the distribution of videos/events with adequately discernible views varied across the 10 individuals labeled as Tak (2 videos), Cedar (5), Utah (12), Ozzy (4), Justin (2), Dame‐Anne (4), Janusz (2), Alan (3), Planck (1), and Pretty (3). To maintain unbiased characterization, grip analysis was performed separately for each algae fishing video, prior to determining which episodes/videos corresponded to the same chimpanzee individual and statistically analyzing the data (see subsection below). Intra‐observer repeatability was confirmed by FAK through repeated descriptions of 10 randomly selected events on a different day, consistently identifying the same grasping patterns (see Results). Overall, despite the inevitable challenges in wild chimpanzee observations related to low video resolution, dynamic gripping actions, and highly variable viewing angles, clearly informative screenshots were captured at key stages throughout the fishing process (including insertion, fishing, extraction, and feeding stages), to identify the key hand components involved. Nevertheless, due to limited video quality and obscured angles of view, not all stages were observable per episode. As such, we opted not to statistically compare grasping patterns across explicit stages in this study and instead encourage future research on wild chimpanzee algae fishing to address this objective based on additional data.

To facilitate description and analysis of algae fishing grips, we divided the observable grasping patterns into two main categories: Group A, which includes grips involving variable thumb–index ray interactions (e.g., pad‐to‐side and V‐pocket configurations), and Group B, which includes *scissor holds* and various hook‐like grips with minimal or no thumb involvement (see Table S1 for details on each grip). This broader grouping relied on the visible morphology of the grips and, importantly, on the functional involvement—or absence—of the thumb ray, which has been highlighted in previous studies as central to stick‐involving manipulative behaviors in chimpanzees (e.g., Marzke et al. 2015; Osuna‐Mascaró et al. 2021). More broadly, these patterns are particularly relevant from an evolutionary perspective, given the fundamental role of thumb‐finger interactions in hominin tool‐related manipulation and their general functional significance in great apes for behaviors requiring fine manipulation. Several variations within each grip group were identified and are named in Table S1, where more explicit definitions and anatomical references are provided. Additionally, visual examples of each grip identified within Groups A and B are provided in the Results.

Whilst observing grips, we noticed that chimpanzees frequently secured and manipulated the end of the stick within their palm and/or fingers (e.g., Bardo et al. 2017; Corp and Byrne 2002; Marzke et al. 2015; Marzke and Wullstein 1996), using this portion of the stick as some form of handle. This tendency broadly resembles certain human‐like forms of precise tool handling, primarily involving the thumb and index finger rays, which enhance the ability to adjust the exact orientation of small gripped objects (Marzke 1997). To our knowledge, this tendency has not been explicitly described in previous observational studies of wild chimpanzee behaviors involving sticks (i.e., approximately cylindrical and fragile objects with a thin diameter, which can often require precise handling), even though it appears to be depicted in illustrations of previous research (e.g., in figure 5 of Osuna‐Mascaró et al. 2021). To further explore this trend for algae fishing, we subdivided Group A's grips into *protruding* (where the stick protrudes from the back of the hand) and *within‐hand* (where the stick's end remains manipulated within the palm and/or fingers). It should be noted that the term *within‐hand* in this paper specifically refers to manipulation of the stick's end (i.e., the “handle”) inside the hand, and does not correspond to the broader *in‐hand* definition of repositioning an object within the hand (Bardo et al. 2017; Elliott and Connolly 1984), which is not necessarily restricted to whether the stick's end protrudes. While the stick's end was also occasionally held *within‐hand* in Group B's *transverse hook* grips (Table S1), it remained *protruding* in the large majority of observed cases (14/20). This pattern aligns with the mechanical demands for stability and leverage required by this grip, particularly during actions such as fishing (involving rotating the stick in the water) or extraction (lifting stick and algae out of the water), and especially more so when using partially flexed fingers. For the *scissor hold* grip, the total number of identified cases was very low overall. In this framework, to avoid overcrowding our charts and increase clarity in the analyses, we opted not to distinguish between these two modes for the grips of Group B. Finally, it should be emphasized that we do not claim this list of identified grips to be exhaustive for chimpanzee algae fishing, as many recorded grasping patterns were ambiguous due to video resolution and/or angle of view.

### 2.5 Data Analysis

All statistical analyses were conducted in R using version 4.4.1. For each behavior and individual a handedness index (HI) was calculated with the formula (*R*−*L*)/(*R* + *L*) whereby *R* equals the number of tool use episodes or throws where the tool is controlled by the right hand of the chimpanzee, and *L* equals the number of tool use episodes or throws where the tool is controlled by the left hand. HI can range from +1 (completely right‐hand biased) and −1 (completely left hand biased). Given the variation in observations per individual, we did not calculate individual HI scores for individuals with fewer than six episodes available, a standard used in previous studies (Marchant 2015). We also calculated the mean HI for each behavior along with the standard error of the mean (SEM). We conducted one sample *t*‐tests to examine if there was any bias in handedness at the behavior level whereby the null hypothesis is that mean HI = 0. Given the very small sample size of individuals for nut cracking we combined the data from the two groups. The *t* statistic was calculated as *t* = (mean HI‐0)/SEM and *p* values assessed for a two‐tailed test given *N*−1 degrees of freedom using the function ‘tdist’ in MS Excel version 2501. Due to deviations from normality and low sample sizes for some of the behaviors we additionally ran non‐parametric Kruskal‐Wallis test (using built in R function ‘kruskal.test’) to examine if individual HI across the three behaviors differed significantly.

We further fitted a general linear model (GLM) to examine the effect of behavior and sex on the likelihood of right‐handedness. We used one data point per individual chimpanzee due to the difficulties of fitting repeated observations with a complex random effect structure of event ID and site ID across behaviors. We included individuals whose hand preference was the left or right hand (*N* = 20/21 chimpanzees). The GLM was fit using the function ‘glm’ from the R package *lmer* with a binomial error structure and logit link function. The response was whether the individual was right‐handed (y/n) and the tool use behavior and sex were fitted as main predictor variables. No interaction between behavior and sex was possible given that all AST throws were by males.

To examine the algae fishing behavior in more depth given the robust sample size of data, we analyzed whether the episode duration of algae fishing was influenced by the handedness or sex of the tool user. Here only complete episodes (start and stop observed to calculate duration) were included in the analysis. We fit an interaction between the two main predictors first, and we included event ID and individual ID as random effects in the model. The linear mixed model (LMM) was fit using the function ‘lmer’ from the *lmer* package in R with a gaussian error structure and identity link function. For both statistical models, a full versus null model comparison whereby the null model had no predictor variables (only the random effects if applicable) was conducted using a likelihood ratio test with the function ‘anova’ in R. If this showed significance, we examined the effect of individual predictors using the ‘drop1’ function in R which also performs likelihood ratio tests to assess significance. We further ensured collinearity was not an issue for our models by calculating variance inflation factors using the package *car* in R (highest VIF in either model was 1.18). We also visually examined plots of residuals to ensure they were normally distributed for the lmm, and that both models were stable (coefficients did not change significantly) when individuals or events were removed one at a time.

Lastly, grasping variability during algae fishing was assessed using both visual and statistical approaches. First, the relative frequency of the observed grips in the 10 analyzed individuals was summarized using a ring plot (calculated based on the total occurrences of each grasping pattern across all videos), accompanied by example screenshots for each grip. The ring chart was generated based on the total number of videos clearly showing each grip variation performed. Additionally, another two ring plots were generated for each hand preference separately. This approach was preferred over relying on the number of events per grip (see Tables 1 and 2) because some events are represented by multiple videos, even though each of these videos documents distinct algae fishing episodes with their unique set of grasping sequences and contexts (e.g., exact location of the algae and the individual within the scene).

**TABLE 1:** Summary of camera‐trap video data and Handedness Index (HI) scores of 37 wild chimpanzees from 4 sites in West Africa engaged in tool‐use behaviors. HI scores were only calculated for the 21 chimpanzees with a minimum of 6 episodes of observation.

	Site	Chimp ID	Sex	Hand used	HI	Number of tool use episodes^a^	Number of videos	Number of events
Algae Fishing	Bakoun	Pretty	Female	Right	1	102	33	9
	Bakoun	Beatrice	Female	Left	−1	31	11	4
	Bakoun	Charlotta	Female	Left	−1	40	6	1
	Bakoun	Dame_Anne	Female	Right	1	38	17	5
	Bakoun	Dawna	Female	Left	−1	7	3	3
	Bakoun	Thunberg	Female	Left	−1	6	2	1
	Bakoun	Alan	Male	Right	1	101	33	7
	Bakoun	Cedar	Male	Left	−1	174	37	8
	Bakoun	Desmond	Male	Right	1	10	4	2
	Bakoun	Janusz	Male	Right	1	13	7	5
	Bakoun	Justin	Male	Left	−1	55	16	7
	Bakoun	Maurice	Male	Left	−1	11	3	2
	Bakoun	Ozzy	Male	Right	1	89	26	6
	Bakoun	Planck	Male	Left	−1	61	13	4
	Bakoun	Tak	Male	Right	1	17	7	3
	Bakoun	Utah	Male	Left	−1	100	25	2
Accumulative Stone Throwing (AST)	Boe	Blotchy	Male	Left	−1	6	6	6
	Boe	Butterfly	Male	Right		1	1	1
	Boe	Chip	Male	Right		1	1	1
	Boe	Dot	Male	Left		4	3	1
	Boe	Dwight	Male	Left		1	1	1
	Boe	Hollow	Male	Left		1	1	1
	Boe	Leftcut	Male	Left		1	1	1
	Boe	Patch	Male	Right		1	1	1
	Boe	Rightcut	Male	Ambi^b^	0.14	7	6	6
	Boe	Righty	Male	Right	1	8	8	4
	Boe	Straightbrow	Male	Left		1	1	1
	Boe	Triangle	Male	Left		1	1	1
Nut cracking	Djoroutou	Anne	Female	Right		4	3	3
	Djoroutou	Alexis	Female	Right	1	9	6	2
	Djoroutou	CSFem17	Female	Left		2	1	1
	Djoroutou	CSFem20	Female	Left		2	2	2
	Djoroutou	CSFem27	Female	Right		2	1	1
	Djoroutou	Dimi	Male	Right		1	1	1
	Sapo	DBFem04	Female	Right	1	12	7	1
	Sapo	Funny face	Male	Left		1	1	1
	Sapo	Pushkin	Male	Right		2	1	1

^a^ For AST = number of throws.

^b^ Ambi means ambidextrous.

**TABLE 2:** Summary of number of videos, events and videos per grip observed for each algae fishing chimpanzee.

Chimp ID	Hand preference	Sex	Number of videos	Number of events	Group A				Group B	
					Protruding	Within‐hand	Interdigital 2/3 protruding	Interdigital 2/3 within‐hand	Scissor hold	Transverse hooks
Alan	Right	M	3	2	0	2	0	0	2	0
Cedar	Left	M	5	5	2	4	0	0	0	2
Janusz	Right	M	2	2	2	0	0	0	0	1
Justin	Left	M	2	2	1	1	0	0	0	1
Ozzy	Right	M	5	2	1	1	2	4	0	1
Planck	Left	M	3	4	1	1	0	0	0	2
Tak	Right	M	2	1	0	0	1	1	1	1
Utah	Left	M	12	2	6	6	0	0	1	5
Dame‐Anne	Right	F	4	2	1	0	0	0	1	3
Pretty	Right	F	3	3	2	1	0	0	0	0

Furthermore, to statistically explore inter‐individual variation in combinations of different grips and their potential association with hand preference (left or right) and/or sex, we performed a Multiple Correspondence Analysis (MCA), treating all identified grips as categorical binary variables (indicating the presence or absence of a specific grip in each individual's episodes across all its discernible videos; see Table 2) (Tabachnick and Fidell 2012). The analysis was performed using the *FactoMineR* and *factoextra* packages for MCA, along with R's built‐in functions for additional calculations and data processing. The resulting axis scores were plotted in a figure, with hand preference and sex color labeled. To facilitate interpretation of the observed multivariate patterns among individuals, the coordinate values for the “presence” condition (value = “1”) of each grip variable along Axis 1 were also displayed within the figure (using Adobe Illustrator v.2015; Adobe Systems, San Jose, CA). These coordinates were not shown for Axis 2, due to the extensive overlapping across individuals and hand preference levels on that dimension. Finally, to evaluate whether the varying sample size among chimpanzees may have significantly impacted the MCA results, the association between the individuals' number of videos and MCA scores (Axes 1 and 2) was evaluated using a Spearman's rank test and an alpha of 0.05.

## 3 Results

Chimp&See community scientists tagged 18 videos from Sapo for nut cracking tool use (e.g., Video S1), 170 videos from Djouroutou for nut cracking tool use, and 312 videos from Bakoun for algae fishing tool use (e.g., Video S2). At Boe, there were 47 different throws by chimpanzees recorded on 56 videos at 9 AST trees (camera‐traps were paired at AST sites so the same throw was at times caught on multiple cameras). We could clearly see and identify the thrower for 33 throws recorded on 31 videos (e.g., Video S3).

Fifty‐eight of the 500 total videos of nut cracking and algae fishing were re‐coded for interobserver agreement tests representing 11.6% of these data (20/212 for Djoroutou nut cracking, 3/18 Sapo nut cracking, 35/312 algae fishing videos). There was excellent agreement between the naïve coder and videos coded by authors AC and SB‐E for type of behavior (*k* = 0.903, *p* < 0.001, *N* = 125) with hand used demonstrating perfect agreement (*k* = 1, *N* = 65). The timing of tool use episodes also showed good agreement, better than expected by chance (start times: *p* < 0.001, *N* = 135; stop times: *p* < 0.001, *N* = 83). There was 100% agreement by AK when reviewing JW's coding of hand used for AST throws across the 31 videos (*N* = 33).

All throws and algae fishing were done with a single hand holding and controlling the tool during the primary tool use action (fishing or throwing). For nut cracking, whenever the hammer was particularly large and heavy, two hands were used to hold and use the hammer during pounding, but otherwise all individuals used a single hand. We excluded all two handed/bi‐manual nut cracking episodes when calculating HI. We were alert to potential switching of hands during tool‐use episodes but did not observe this for algae fishing nor nut cracking. Of the 37 chimpanzees in this study, 21 had more than 6 tool‐use episodes available for an HI score to be calculated (Table 1).

Twenty of the 21 chimpanzees with sufficient observations demonstrated a preference for the left (HI = −1) or right hand (HI = 1) for the tool‐use behaviors examined. There was one AST chimpanzee who was observed using both the right and left hand to throw stones at trees (Table 1). Of the 21 chimpanzees, nut cracking had 2 right‐handed: 0 left‐handed chimpanzees; algae fishing 7:9 and AST 1:1 with 1 ambidextrous individual. The mean HI and standard error of the mean (SEM) for nut cracking was 1.00 ± 0; for algae fishing −0.125 ± 0.26; and for AST 0.047 ± 0.58. A one sample *t*‐test was nonsignificant, supporting no significant behavior‐level bias in handedness for algae fishing (*t* = −0.49, *p* = 0.63, df = 15), and due to the low sample size, *t*‐tests were not conducted for nut cracking or AST. A non‐parametric Kruskal‐Wallis test was also nonsignificant (*χ* ^2^ = 2.25, df = 2, *p* = 0.32) supporting no differences in HI according to tool‐use behaviors. The GLM examining effects of tool use behavior and sex on the probability for individuals to be right‐handed was also not significant from the null model (*χ* ^2^ = 3.45, *p* = 0.33, *N* = 20).

The LMM testing for variation in algae fishing duration as a function of preferred hand and sex of tool user showed a weak trend for the interaction between the two predictors (*χ* ^2^ = 2.84, *p* = 0.09, *N* = 657). We therefore tested the main effects model whereby the full versus null model comparison was significant (*χ* ^2^ = 7.70, *p* = 0.02, *N* = 657). Right‐handed individuals spent more time algae fishing (estimate ± SE: 3.74 ± 1.20, *t* = 3.13, *p* < 0.006; Figure 1) while sex had no significant effect (estimate ± SE: 0.32 ± 1.28, *t* = 0.25, *p* = 0.81). Fishing duration varied considerably across algae fishing episodes with a mean of 8.47 s (range: 1.07–37.55 s).

**FIGURE 1:**
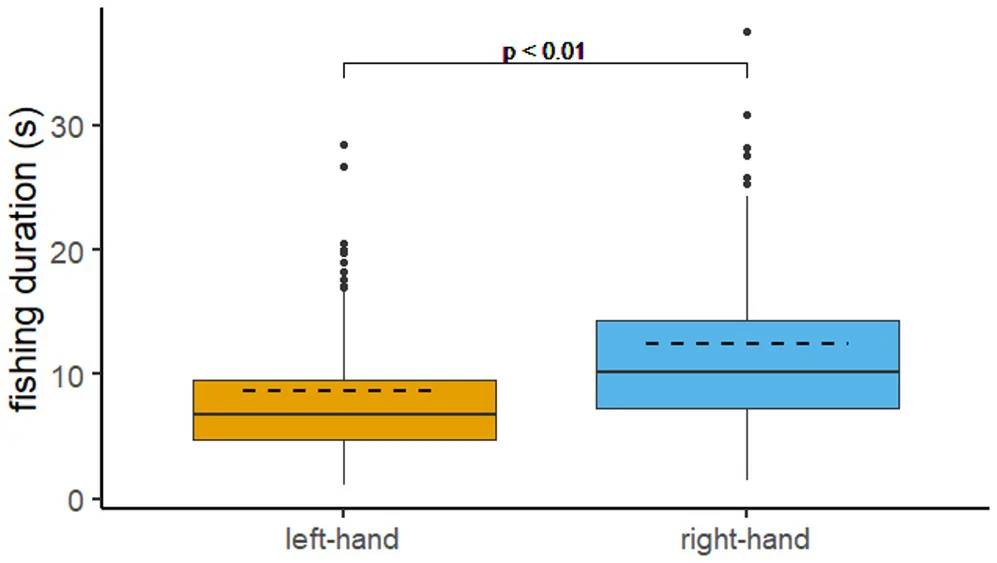
Boxplot of algae fishing duration by chimpanzees that consistently used their left or right hand (*N* = 657 episodes from 16 chimpanzees). The box shows the median value of the raw data (*horizontal black line*) whilst the model line (*dashed horizontal line*) shows the model‐derived estimate. The vertical whiskers show the 1st and 3rd quartiles of the data.

Our video observations revealed remarkable variability in grasping patterns during algae fishing. As explained above in Materials and Methods, we classified these grasping tendencies into two broader grip categories (i.e., Group A and Group B), each further divided into several variations (Table S1). Grip patterns per individual are summarized in Table 2. A ring plot displays the relative frequency of each identified grasping pattern in the entire sample (Figure 2), while the two ring plots of Figure 3 provide the same information per hand preference group (left or right). The latter shows certain grasping similarities between left‐ and right‐handed individuals (mainly the similar representation of the broad category of *transverse hooks*; see Figure 3; Table 2), but also substantial differences: left‐handed individuals show a higher representation of precise Group A grips (both *protruding* and *within‐hand*), while right‐handed chimpanzees more frequently use *Interdigital 2/3* grasping (*protruding* and *within‐hand*) patterns.

**FIGURE 2:**
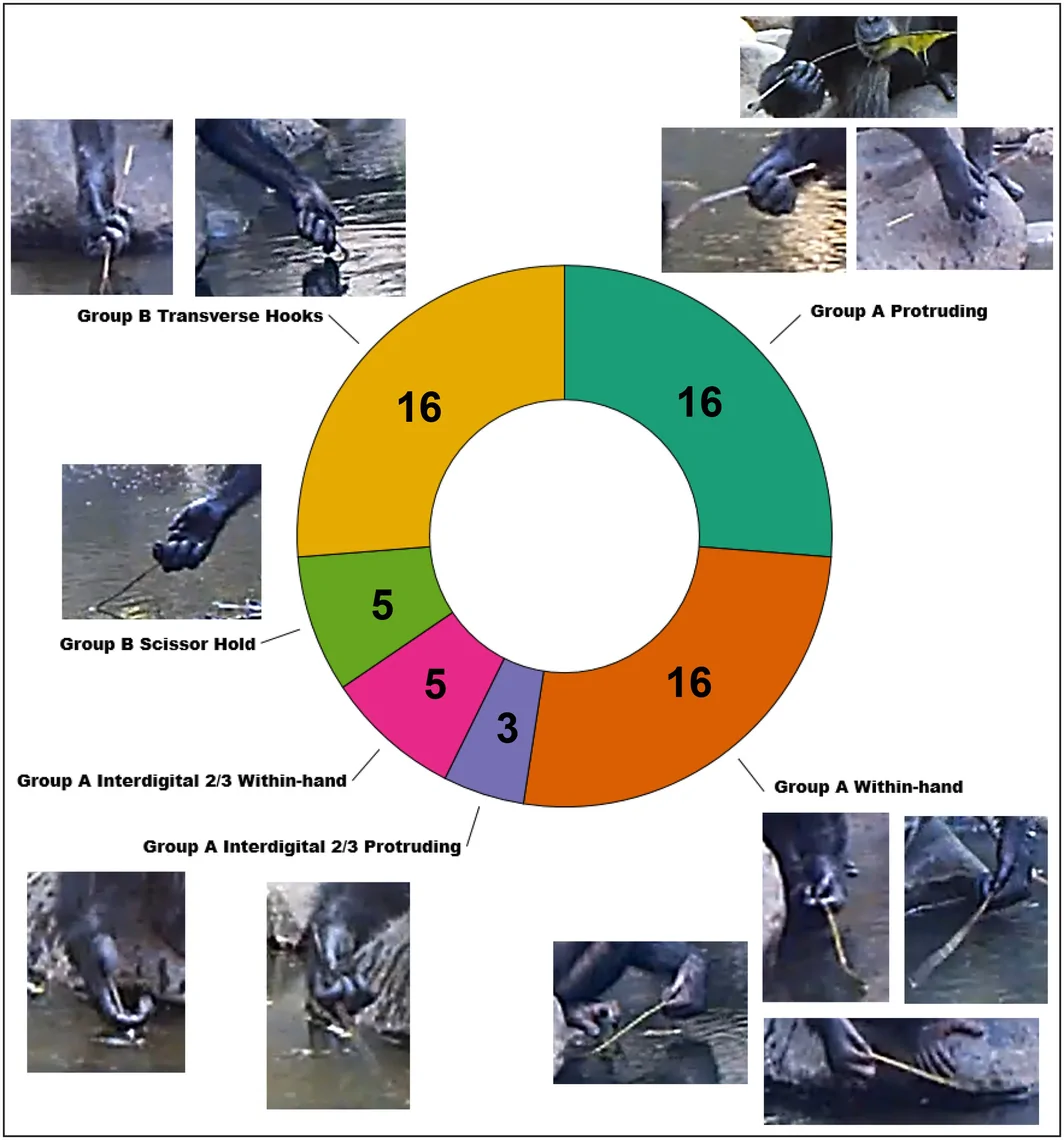
Ring plot showing the relative frequency of each identified grasping pattern for algae fishing across the sample of 41 videos (for definitions, see Table S1), with example screenshots from the data.

**FIGURE 3:**
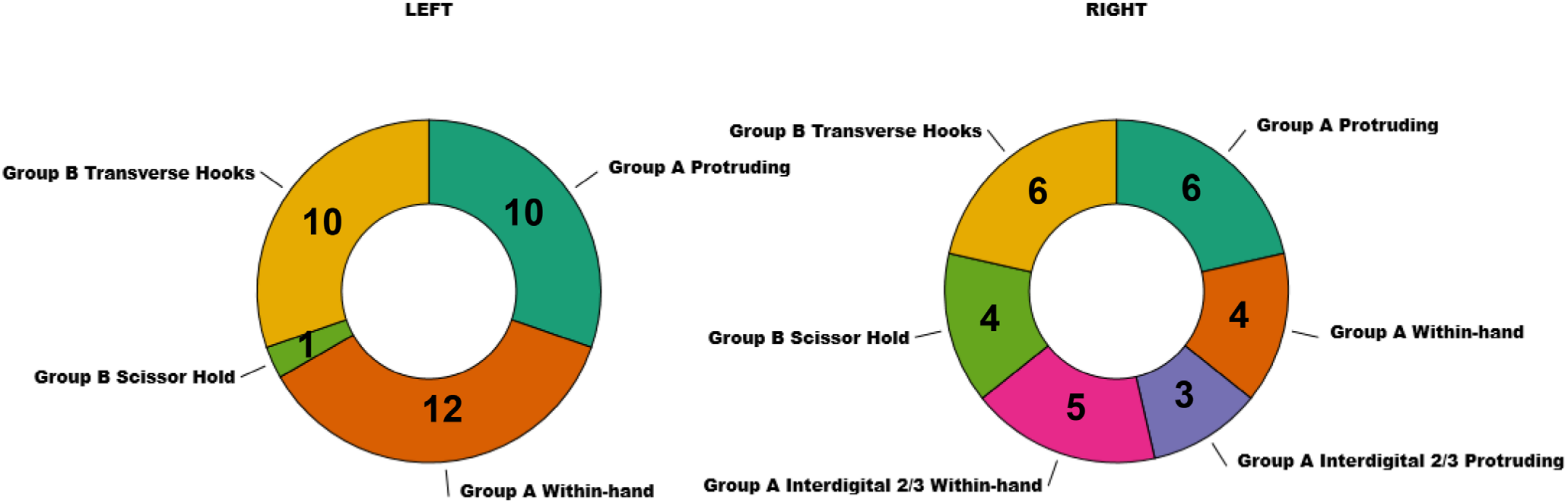
Ring plots showing the relative frequency of each identified grasping pattern for algae fishing across videos per hand preference (left‐ or right‐handed).

The MCA plot (Figure 4) provided additional insights into these interindividual trends. Along Axis 1, as reflected on its variable coordinate values, individuals with positive scores displayed relatively more frequent *interdigital 2/3* grips, a pattern particularly represented by two male adult chimpanzees, while individuals with negative scores tended to favor more the thumb‐involving Group A grips. The near‐zero loading of the broad *transverse hook*s (see Table S1) is consistent with the fact that this grasping trend was utilized by most individuals in this sample (see Figure 2 and Table 2). Interestingly, individuals who utilized *within‐hand* grips (where the stick's end is manipulated within the palm and hand) also tended to use the corresponding *protruding* grips (where the stick's end protrudes from the back of the palm), suggesting that these two modes were similarly frequent within individuals. In terms of grasping trends by hand preference, the MCA plot (Figure 4) underscores a notable clustering pattern: all left‐handed individuals (in orange) clustered within the negative Axis 1 side, primarily associated with relatively more frequent thumb‐involving Group A grips. In contrast, right‐handed individuals (in light blue) displayed a much larger spread across both axes, indicating a much greater variability in grasping combinations. This pattern may hint at a more consistent use of thumb‐involving precision grips among left‐handed individuals, yet caution is warranted given the limited sample size (see Discussion below). The Spearman's test found no significant correlation between the number of videos analyzed per individual and the scores of Axis 1 (*p* = 0.90) and Axis 2 (*p* = 0.92).

**FIGURE 4:**
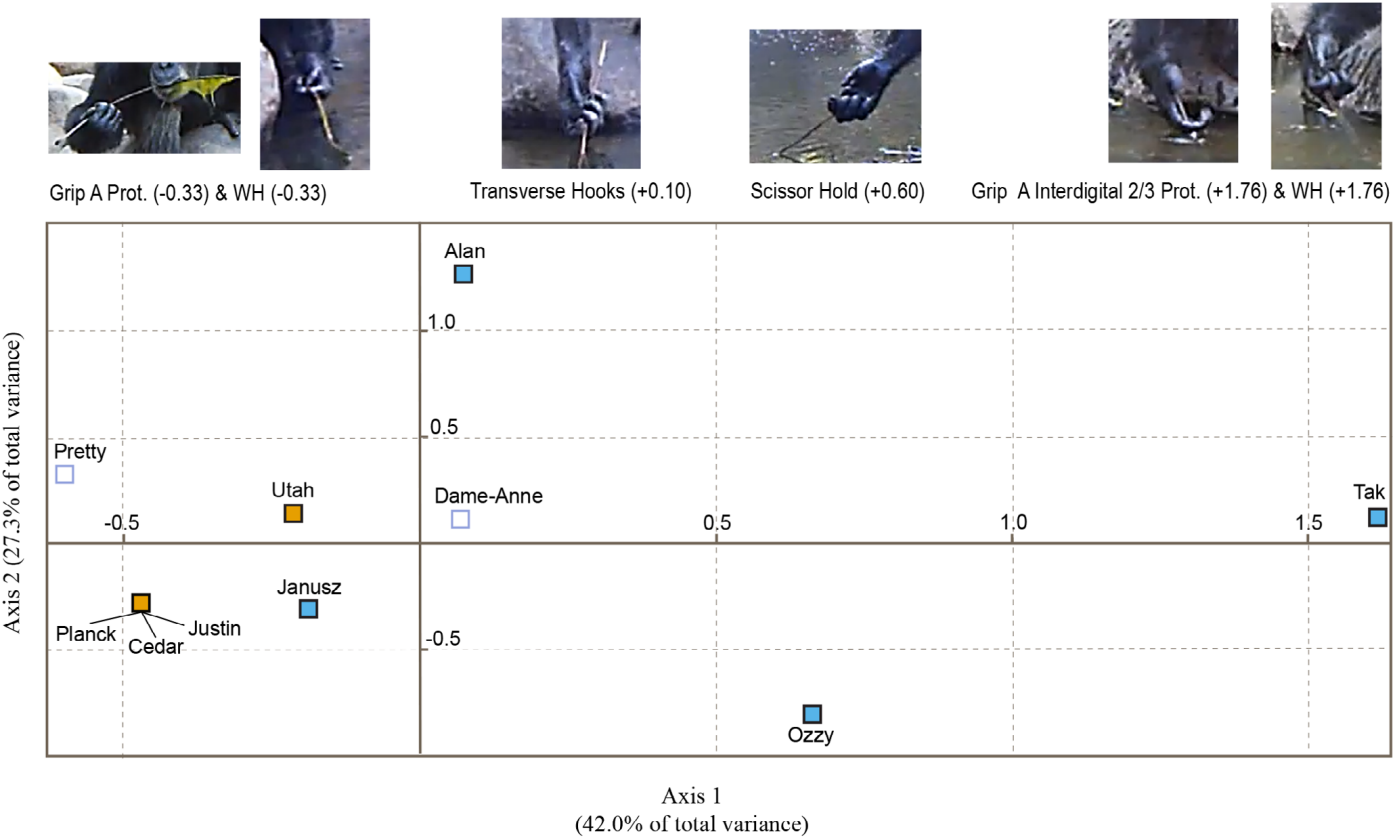
Multiple Correspondence Analysis (MCA) plot of Axis 1 and 2, illustrating inter‐individual variability in grasping combinations. Left‐handed individuals are color‐coded in orange, while right‐handed individuals are shown in light blue. Female individuals are represented by open square symbols. At the top, the coordinate values on Axis 1 for the presence condition (value = 1) of each grip variable are displayed, alongside example images of the corresponding grips.

## 4 Discussion

Twenty of the 21 individual chimpanzees with sufficient observations in this study consistently used either the left or right hand for unimanual tool‐use tasks: algae fishing, nut cracking, and accumulative stone throwing (AST). There was one ambidextrous individual who was recorded throwing stones with both the left and right hand at AST trees. These results provide critical insight into the degree of individual laterality in hand‐use observed across wild chimpanzee tool‐use behaviors. In previous studies, *complete* lateralization or exclusive hand preference, where an individual *always* uses either the left or right‐hand for a task, is often a small fraction of individuals observed (e.g., 3/17 chimpanzees for termite fishing in Lonsdorf and Hopkins 2005; 12/27 for termite fishing in Bogart et al. 2012; 0/16 chimpanzees for unimanual throwing in Nishida et al. 2012). However, similar to our findings, there are studies that report a large number of wild chimpanzees being *consistently* lateralized to the left or right (e.g., 55/89 chimpanzees for termite fishing in Sanz et al. 2016; 12/15 chimpanzees for nut cracking in Sugiyama et al. 1993; 18/22 for nut cracking in Humle and Matsuzawa 2009). There are also some studies that report circa 50% of individuals completely lateralized to the left or right (18/36 for termite fishing in McGrew and Marchant 1999; 18/41 for nut cracking in Boesch 1991; 9/14 for anvil use for *Strychnos* pounding in McGrew et al. 1999). Therefore, our observations of complete lateralization for all but one of the 21 chimpanzees in this study are not without precedent, which is reassuring given the low number of observations for nut cracking and AST. It is important to note that previous studies have sometimes included young or juvenile individuals which could affect the degree of lateralization for an individual given ontogenetic development of tool use techniques and handedness (Boesch 1991; Humle and Matsuzawa 2009). Another potential reason for why we have detected such strong hand preferences for each individual may also be due to the very narrow set of conditions for each tool use behavior we examined. For example, we investigated throwing in a highly specified context where chimpanzees unimanually throw stones at AST trees from a relatively close proximity.

Similar to previous studies (Boesch 1991; Humle and Matsuzawa 2009; Lonsdorf and Hopkins 2005), we found no statistically significant sex differences in hand preference for these tasks. We also found no statistical evidence for behavior‐level handedness biases. This is similar to previous studies on nut cracking (Boesch 1991; Sugiyama et al. 1993), termite fishing (McGrew and Marchant 1992, 1999; Sanz et al. 2016), and throwing (Nishida et al. 2012). However, when studies pool sample sizes from more than one group, they report population‐level left hand bias for termite fishing (Bogart et al. 2012) and a population‐level right hand bias in nut cracking (Lonsdorf and Hopkins 2005). Indeed, the two individuals with sufficient observations for nut cracking were both right‐handed. Algae fishing, similar to algae scooping (Humle and Matsuzawa 2009), tended to have a small mean HI close to the null hypothesis of 0, similar to AST. We must acknowledge that our low sample sizes, specifically for nut cracking and AST, may be too low to draw any strong conclusions regarding HI scores and exclusive hand preference. In particular, we may be underestimating the number of ambidextrous individuals; therefore, more data from these communities would be helpful. It would also be useful to examine these behaviors across multiple years to determine if hand preference remains the same across individuals. However, previous research on termite fishing and nut cracking would suggest that to be highly likely once individuals reach adulthood (Biro et al. 2003), and all the chimpanzees in our sample were adolescents or adults.

Our study is one of few to examine tool‐use task related performance in relation to hand preference in wild chimpanzees. Sanz et al. (2016) measured performance asymmetries for termite fishing whereby right‐handed individuals had faster (shorter) dipping durations than left‐handed individuals which was assumed to represent greater efficiency. Also, for termite fishing, McGrew and Marchant (1999) showed that lateralized individuals were more efficient at gathering termites than individuals that did not show a clear hand preference. Here we found that right‐handed algae fishers had longer fishing durations than left‐handed individuals which may result in meaningful variation in the ability to successfully extract algae. Either right‐handed individuals are able to fish for longer and extract more algae as a result in comparison to left‐handed individuals, or they extract less and are actually less efficient because it takes them longer. Based on our observations of the behavior we would expect it is the former, given that we often observe individuals capturing large amounts of algae on their stick only after having the stick submerged for a good amount of time. Large amounts of algae may also require more time for gathering it because the algae has to be balanced on the end carefully to prevent it from slipping off prematurely. We also observed that for many of the algae fishing locations it appears difficult to balance at the water's edge while fishing. Chimpanzees are often in a bent tripedal or even bipedal stance while holding the stick in one hand for fishing and stretching their arm out to reach some distance into the water. Hence, individuals that can fish for longer may be better at balancing. Data on actual algae amounts extracted as well as controlling for aspects such as particular algae fishing location would be helpful in teasing out the effects of this performance asymmetry on algae fishing success.

Grasping patterns involved in algae fishing were in line with our expectations whereby the identified grips closely resemble those reported in previous research on wild chimpanzee behaviors involving stick tool use (Lesnik et al. 2015; Malherbe et al. 2024; Marzke et al. 2015; Marzke and Wullstein 1996; Osuna‐Mascaró et al. 2021). For instance, the consistent use of precise thumb‐index interactions, primarily pad‐to‐side grips exerting relatively low force, aligns with findings from Marzke et al. (2015), who reported that these grips accounted for 90% of all identified patterns when manipulating thin and cylindrical ant‐dipping tools, with the remaining 10% corresponding to index finger probe movements. Additionally, the frequent use of more forceful *transverse hook* grips and *interdigital 2/3* braces observed in our study has also been highlighted in previous research on stick tool use for termite nest perforation and fishing (Lesnik et al. 2015; Osuna‐Mascaró et al. 2021). These grips likely facilitate movements requiring relatively higher levels of force compared to the above‐mentioned thumb‐index interactions (Lesnik et al. 2015; Marzke 1997). Overall, our findings suggest that algae fishing behavior combines thumb‐involving precision (Group A grips) as described by Marzke et al. (2015) with more forceful grips relying on hook and interdigital braces (Lesnik et al. 2015; Osuna‐Mascaró et al. 2021). This combination reflects the well‐documented ability of chimpanzees to efficiently adapt their grasping patterns to the size and mass of manipulated objects (Boesch and Boesch 1993; Marzke and Wullstein 1996).

In general, algae fishing seems to likely demand a combination of precision (i.e., aiming and accounting for various positional and environmental factors) and controlled force exertion. When conditions allowed it, the precise and low‐force handling of sticks relying on the thumb and index finger (Group A *protruding* and *within‐hand*) was sufficient. However, when it was required (e.g., due to factors like the distance to the target, water depth, or the size and angle of algae relative to the stick's length and diameter), more forceful grips, such as *transverse hooks* (including extended and diagonal types) or *interdigital 2/3* braces, were effectively employed. These alternative grips likely provided adequate reinforcement for this objective without necessitating two‐handed manipulation.

The striking interindividual variation in grasping patterns observed in our analyses may reflect low‐fidelity social learning or self‐learning processes, wherein individual chimpanzees develop their own grasping styles according to their unique biomechanical characteristics (e.g., body size) and prior practical cumulative experience. Such individualistic approaches to algae fishing would suggest that this behavior does not require high‐fidelity social transmission of exact techniques and grasping strategies, which would presumably lead to more homogeneous patterns across individuals of a group (Whiten et al. 2005). However this would not preclude other aspects of the tool‐use behavior from being socially learned, such as what and where to fish (Bandini et al. 2020). Further research is needed to elucidate those potential social learning mechanisms.

While our results suggest potential grasping differences related to hand preference, specifically, a greater tendency for thumb‐involving precision grips among left‐handed individuals, it is essential to interpret these findings with caution due to the very small sample size. In particular, only two of the 10 individuals exhibited high Axis 1 scores, only two individuals in the sample were females, and only a single individual showed a distinctive Axis 2 score. As such, the observed trends by sex and hand preference may not necessarily generalize across larger or more diverse populations. Further studies involving greater numbers of individuals and varying environmental contexts would be valuable to assess the robustness of these preliminary observations.

From an evolutionary perspective, the precision handling patterns observed in algae fishing broadly resemble certain human‐like manual strategies for securing elongated tools within the hand, largely relying on fine and variable thumb‐index finger interactions (Marzke 1997; Marzke et al. 2015; Marzke and Wullstein 1996). This is particularly evident in grips where the relatively thin and fragile stick is secured in a way that allows for controlled and precise movements, similar to the human‐like fine handling of proportionally elongated objects consisting of distinctive handles. Importantly, similar thumb‐index‐palm interactions have been observed in experimental studies on the key biomechanical requirements of early hominin stone tool use, highlighting their critical role in precision manipulation (Eteson et al. 2024; Karakostis, Haeufle, et al. 2021; Key et al. 2020; Marzke et al. 1998). We have further observed a similar grasping pattern for underground termite fishing in chimpanzees of the Wonga Wongué Presidential Reserve in Gabon, where the end of long sticks are securely held in the palm for control and precision (Boesch et al. 2020). Although several hand anatomical differences between *Pan* and *Homo* inevitably result in clear functional and biomechanical discrepancies in the exact performance of these grips (e.g., see Diogo et al. 2012; Karakostis, Haeufle, et al. 2021; Kunze et al. 2024; Marzke et al. 1998), the presence of such precise handling of thin elongated objects in wild chimpanzees reflect a shared capacity with modern humans, presumably inherited from a common ancestor. This evolved capacity might have been gradually enhanced in the hominin lineage, as improved thumb‐based precision handling likely conferred evolutionary advantages related to skilled human‐like manipulation of tools, regardless of their material (wood, bone, or stone).

An additional evolutionary context is provided via comparisons with other great apes, particularly gorillas. Although they rarely use tools in the wild, previous studies on gorillas have shown that they employ complex and, in some cases, similar grasping patterns during food processing to those documented here, as well as to those observed in humans (e.g., Grueter et al. 2013). Notably, in gorillas, the thumb is reported to play a key role in the majority of food‐processing grips, often assisting in forceful manipulation by providing pressure and stability (e.g., Neufuss et al. 2019). This is consistent with their overall hand anatomy which includes a relatively longer and more robust thumb compared to chimpanzees, and supports the hypothesis that great apes early on developed the capacity for high‐precision manual behavior in response to the demands of their foraging niche (Neufuss et al. 2019). This may have provided a fundamental substrate for the later development of human‐like stone knapping abilities. Such underlying capacities may have been further elaborated in other species with the emergence of more refined precision handling and in‐hand manipulation of objects and tools; patterns that were not observed in mountain gorillas (Neufuss et al. 2019), but have been documented in western lowland gorillas, chimpanzees, and bonobos (e.g., Bardo et al. 2017; Crast et al. 2009; see also grips described in the present study).

The above scenario aligns well with the recently emphasized hypothesis of a “Botanic Age” in early human evolution, preceding the advent of systematic stone tool use (Falk 2024). During this hypothetical period, it is proposed that hominin dexterity was shaped by plant‐based tool use (presumably mainly involving sticks), which likely demanded precise manipulation for efficiently extracting food from challenging contexts, such as water or dense/hard soil. Although plant‐based material evidence is inherently difficult to trace in fossil hominin contexts, there are indirect indications of similar behaviors, such as the proposed termite foraging with bone tools by *Paranthropus* about 1.8 million years ago in southern Africa (albeit already within the stone tool era and not necessarily involving wooden sticks) (Backwell and d'Errico 2008). On this basis, it is conceivable that a pre‐stone‐tool “Botanic Age” (Falk 2024) marked a pivotal stage in the evolution of manual dexterity following the *Pan*‐*Homo* split, laying the groundwork for more complex precision grips required for human‐like stone tool production and use to evolve. In this framework, perhaps the challenges posed by algae fishing, termite fishing and other plant‐based tool use behaviors might have favored the evolution of fine motor skills and sophisticated coordination. In this sense, such manual behaviors might have theoretically served as an evolutionary precursor to the manual skills needed for human‐like stone tool production and use (through exaptation). To further explore these possibilities, future studies should include quantitative metrics and analyses to investigate individual stick tool‐use techniques, not only in chimpanzees but also in other dexterous primates (e.g., macaques, capuchins, as well as the other great apes). Additionally, future paleontological and paleoanthropological research comparing extant primate and fossil hominin morphology (e.g., see (Dunmore et al. 2023; Karakostis, Reyes‐Centeno, et al. 2021; Kivell 2015; Kivell et al. 2023; Kunze et al. 2024) and references therein) could focus on identifying skeletal features that reflect the performance of these manual behaviors and identified grasping patterns. Such analyses could eventually help reconstruct the evolutionary history of these grips and their implications.

## Author Contributions

**Ammie K. Kalan:** conceptualization, investigation, funding acquisition, writing – original draft, methodology, writing – review and editing, formal analysis, data curation, supervision, resources, visualization, project administration. **Mimi Arandjelovic:** funding acquisition, writing – review and editing, data curation, resources. **Hjalmar Kuehl:** funding acquisition, writing – review and editing, resources, data curation. **Skye Bartel‐Ens:** writing – review and editing, data curation. **Amy Christensen:** writing – review and editing, data curation. **Julia Whelan:** writing – review and editing, data curation. **Anthony Agbor:** writing – review and editing, data curation. **Paula Dieguez:** writing – review and editing, data curation. **Juan Lapuente:** writing – review and editing, data curation. **Amelia C. Meier:** writing – review and editing, data curation. **Fotios Alexandros Karakostis:** writing – review and editing, writing – original draft, conceptualization, investigation, methodology, visualization, formal analysis.

## Funding

Funding for the Pan African Programme: The Cultured Chimpanzee (PanAf) was provided by the Max Planck Society Innovation Fund, Heinz L. Krekeler Foundation, and Max Planck Society. Funding for Guinea‐Bissau field work by A.K.K. was also supported by the Max Planck Society and a National Geographic Explorers Grant (GEFNE171‐16). A.K.K. also acknowledges funding from the Deutsche Forschungsgemeinshaft (DFG) Centre for Advanced Studies “Words, Bones, Genes, Tools” for a fellowship that supported part of this research project.

## Ethics Statement

The data used for this study are from the Pan African Programme: the Cultured Chimpanzee and associated projects where data collection protocols were approved by the Max Planck Institute for Evolutionary Anthropology and by the respective government ministries that gave permissions for this field work to be conducted (see Acknowledgements for full list of ministries).

## Conflicts of Interest

The authors declare no conflicts of interest.

## Supporting information


**Data S1:** Supporting Information.

**Table S1:** Description of the grasping patterns observed in the videos during wild chimpanzee algae fishing (see also example images in Figures 2 and 4).

**Video S1:** Chimpanzee nut cracking with a stone hammer at Sapo, Liberia. Credit PanAf/MPI EVA.

**Video S2:** Chimpanzees algae fishing at Bakoun, Guinea. Credit PanAf/MPI EVA.

**Video S3:** Chimpanzee accumulative stone throwing at Boe, Guinea‐Bissau. Credit Kalan.

## Data Availability

The behavioral data for this study have been provided as a Supporting Information (Data S1) while the figures and statistical analyses of the grips relied on the data presented in Table 2 of the main text.
