# Supplementary material for: Sticks and Stones: Hand Preference and Grip in Wild Chimpanzee Tool‐Use
Source: Am J Biol Anthropol. 2026 Oct 10;191(2):e70364. doi: 10.1002/ajpa.70364 (PMC13653221; doi:10.1002/ajpa.70364)
Supplement: Supplementary file 1 [file AJPA-191-e70364-s004.docx]

**Table S1.** Description of the grasping patterns observed in the videos during wild chimpanzee algae fishing (see also example images in Figures 3 and 4)

| **Grip Categories** | **Divisions** | **Description** | **Important Considerations** |
| --- | --- | --- | --- |
| **"Group A": Variable Precise Thumb-Index Ray Interactions** | **Group A - Protruding** (i.e., Stick's end clearly protruding from the back of the palm) | Key role of thumb and index rays (including the palm), typically with 2-jaw chuck pad-to-side variations (e.g., Marzke et al., 2015). This is also often combined with variable contributions of the remaining palm and the other 3 rays - depending on direction angle and stick length being handled. | In addition, these grasping patterns may also partly involve the side and tip of the thumb as well as the pad of the index (see Marzke et al. 2015). In some cases, the grip resembles the “Extended V-pocket” configuration involving the fleshy web between the thumb and the index finger, with support from the palm (see Osuna-Mascaro et al., 2020). The visible length of the stick protruding from the back of the palm can vary greatly (on some occasions, only slight). |
|  | **Group A - Within-hand Stick's End** | Stabilization of the stick's end within the palm and fingers (typically relying on pad-to-side combinations), apparently increasing directional control for the selected stick. | Manipulation of the stick’s end primarily with the thumb, index, and sometimes the middle finger rays (including their associated palm tissues), often relying on variable “pad-to-side/pad”, "V-pocket" or "Extended V-pocket" combinations (e.g., Marzke et al., 2015; Osuna-Mascaro et al., 2020). |
|  | **Group A - Interdigital 2/3 brace** | Stick mainly stabilized between fingers 2 and 3, with contribution of the thumb ray's palm region (e.g., Osuna-Mascaro et al., 2020). | **Can be "within-hand"**. |
| **"Group B": *Hooks and Scissors*** (see Marzke et al., 2015; Lesnik et al., 2015) | **Scissor Hold (2/3)** | Stick held and stabilized between rays 2-3 (no discernible thumb ray contribution), palm may partly support. | Often pronated hand orientation (palm down); Can occasionally be "within-hand". |
|  | **Transverse Hook** | Manipulation with flexed fingers, thumb ray involvement being either clearly absent or minimal/questionable, sometimes combined with an extended index ray or an interdigital 2/3 brace (likely to facilitate stick directional control). | Often supinated hand orientation (palm up). Can sometimes be "within-hand". |
|  | **Extended Transverse Hook** | Full flexion of rays 2 to 5, forming a closed grip with the stick crossing the hand. | Occasionally observed but not analyzed separately in this study, due to video quality issues preventing a clear view. In this study's figures/analyses, these were grouped together with the transverse hooks. |
|  | **Diagonal Hook** | Declining flexion of the rays, creating a diagonal configuration. | Occasionally observed but not analyzed separately in this study, due to video quality issues preventing a clear view. In this study's figures/analyses, these were grouped together with the transverse hooks. |

***Note****: The terminology used is based on a combination of literature references (mainly Marzke and Wullstein, 1996; Marzke et al., 2015; Lesnik et al., 2015; Osuna-Mascaro et al., 2020 and references therein), integrated and enriched with additional observations made during the present study.*
